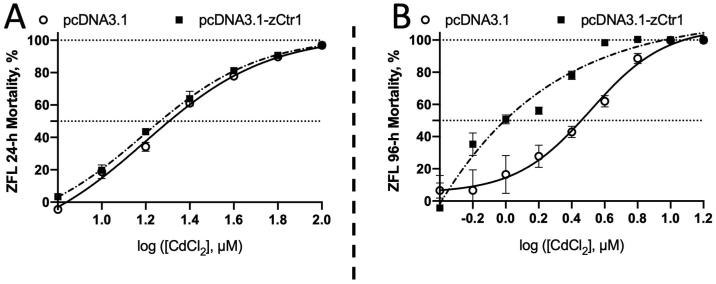# Promotion of cadmium uptake and cadmium-induced toxicity by the copper transporter CTR1 in HepG2 and ZFL cells

**DOI:** 10.1016/j.toxrep.2020.11.005

**Published:** 2020-11-12

**Authors:** Man Long Kwok, Zhen Ping Li, Tin Yu Samuel Law, King Ming Chan

**Affiliations:** School of Life Sciences, The Chinese University of Hong Kong, Sha Tin, N.T., Hong Kong

**Keywords:** h, hours, min, minutes, Cd, Cadmium, Cu, Copper, CTR1, High-affinity Cu-uptake protein 1, hCTR1, Human CTR1 protein, *hCtr1*, Human CTR1 gene, LC_50_, Median lethal concentration, PBS, Phosphate-buffered saline, qPCR, Quantitative real-time PCR, *ybx1*, Y box-binding protein 1 gene, zCTR1, Zebrafish CTR1 protein, *zCtr1*, Zebrafish CTR1 gene, Copper transporter, Cadmium uptake, Cadmium toxicity, Stable cell line

## Abstract

Cadmium (Cd^2+^) is considered a human carcinogen as it causes oxidative stress and alters DNA repair responses. However, how Cd^2+^ is taken up by cells remains unclear. We hypothesized that Cd^2+^ could be transported into cells via a membrane copper (Cu) transporter, CTR1. CTR1 expression was not affected by Cd^2+^ exposure at the mRNA or protein level. Stable cell lines overexpressing either hCTR1, in the human liver cell line HepG2, or zCTR1, in the zebrafish liver cell line ZFL, were created to study their responses to Cd^2+^ insult. It was found that both HepG2 and ZFL cells overexpressing CTR1 had higher Cd^2+^ uptake and thus became sensitive to Cd^2+^. In contrast, hCTR1 knockdown in HepG2 cells led to a reduced uptake of Cd^2+^, making the cells relatively resistant to Cd^2+^. Localization studies revealed that hCTR1 had a clustered pattern after Cd^2+^ exposure, possibly in an attempt to reduce both Cd^2+^ uptake and Cd^2+^-induced toxicity. These *in vitro* results indicate that CTR1 can transport Cd^2+^ into the cell, resulting in Cd^2+^ toxicity.

## Highlights

• CTR1-overexpressing HepG2 and ZFL cell lines were created.
• CTR1 overexpression in both HepG2 and ZFL cells increased Cd^2+^ uptake and toxicity.
• CTR1 knockdown in HepG2 cells decreased Cd^2+^ uptake and toxicity.
• CTR1 plays a significant role in Cd^2+^ uptake and toxicity.

## Graphical abstract

**Figure:**
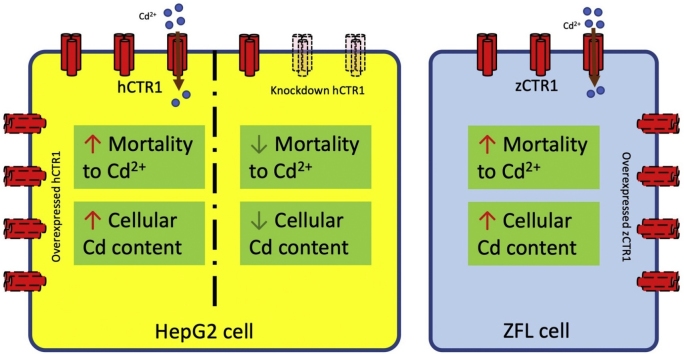

## 1 Introduction

Cadmium (Cd) is a divalent metal with an atomic number of 48. It is normally found in the oxidation state Cd(II) (Cd^2+^), but Cd(I) (Cd^1+^) has also been observed [1]. Cd^2+^ is defined as a transition metal, although some chemists do not regard it as a true transition metal as it has a full shell of d electrons. Cd^2+^; is an excellent electrical conductor and is resistant to corrosion. Cd’s primary use is as a component of rechargeable batteries; however, it contributes to industrial pollution and is bio-accumulative in the human body. A Cd^2+^ bio-accumulative disease called *Itai-itai* disease has been reported in women residing in rice farming areas irrigated by the Cd^2+^-contaminated Jinzu River in Toyama, Japan [2]. Although the marine diatom *Thalassiosira weissflogii* uses cadmium as a catalytic metal atom in cadmium carbonic anhydrase CDCA1 [3], Cd^2+^ has no known function in higher organisms or animals.

Cd^2+^ is classified as a human carcinogen Group B1, probable human carcinogen, by the United States Environmental Protection Agency [4], and as Group 1, carcinogenic to humans, by World Health Organization [5]. Potential factors contributing to Cd^2+^ oncogenicity include induction of aberrant gene activation, suppression of apoptosis or impairment of efficient DNA repair [6]. Cd^2+^ induces oxidative stress by producing free radicals, which significantly increases lipid peroxidation, leading to induction of superoxide dismutase activity [[7], [8], [9]]. Cd^2+^ is a redox-stable metal, meaning that Cd^2+^-induction of free radical production must be mediated through an indirect mechanism, generating free radicals by disrupting cellular antioxidant systems [10]. CdCl_2_ affected mitochondrial function leading to differential production of ATP in media containing glucose or galactose, like Warburg effect in tumor cells [11]. Besides, CdCl_2_ increased the passive tension of trabecular muscle from the right ventricle in rats [12]. CdS nanoparticles can cross the blood-brain barrier in dose-dependent manner, without significant toxicity below 0.01 μg /mL [13].

The high-affinity copper-uptake protein 1 (CTR1) is encoded by the SLC31A1 gene [14]. CTR1 belongs to a family of proteins that provide copper (Cu) for Cu chaperones [15] and is found on the plasma membrane as a triplex [16]. CTR1 is expressed ubiquitously across all eukaryotic cells. It was first identified in yeast [17], with homologues subsequently identified in fish, mammals and humans to transport Cu^+^ across cellular membranes [14,18]. CTR1 is vital to many developmental processes, with embryonic lethality occurring in CTR1 knockout and knockdown mice [19,20] and zebrafish [21], respectively.

HepG2 is a human (*Homo sapiens*) liver cancer cell line. It has been used in several toxicology studies [22,23]. Cd^2+^ was shown to be carcinogenic to HepG2 cells [24], and was found to affect the p53 pathway without inducing the expression of p53 itself [25]. CTR1 has also been studied in HepG2 cells, where it was confirmed as a transporter of Cu [26].

The mechanism of cellular Cd^2+^ homeostasis is still not fully understood. Previous studies have proposed that Cd^2+^ is transported into cells via zinc and iron homeostatic mechanisms, such as ZIP8 and DMT1 [27,28]. Similarly, it has been proposed that its elimination from the cell may occur via Cu transporters, such as the ATP7 family.

Zebrafish is a freshwater fish species of the family Cyprinidae, it is a common model organism for toxicological and biomedical studies with the advantages of its small size, short reproductive cycle and transparent embryos [29,30]. ZFL is a zebrafish (*Danio* rerio) liver cell line previously used to study Cu-induced reactive oxygen species production [31] and Cd-induced cytotoxicity [8]. CTR1 was confirmed to transport Cu in ZFL cells in our previous study [32]. In this study, we investigated a potential cellular uptake mechanism for Cd^2+^. We hypothesized that CTR1 plays a role in Cd^2+^ uptake. We used the human cell line HepG2 as the primary model, in parallel with the zebrafish cell line ZFL as a supplementary model, to determine the relationship between CTR1 and Cd^2+^.

## 2 Materials and methods

### 2.1 Cell culture

HepG2 and ZFL cells are adherent hepatocyte cell lines derived from humans and zebrafish, respectively, and were obtained from the American Type Culture Collection (ATCC, USA). HepG2 culture medium contained Dulbecco’s Modified Eagle Medium (12,100,046; Gibco, Massachusetts, USA). ZFL culture medium contained 50 % l-15 medium (11,415,064; Gibco), 35 % Dulbecco’s Modified Eagle Medium and 15 % Hans F12 (21,700,075; Gibco) with 15 mM HEPES (11,344,041; Gibco). Both culture media were supplemented with 0.15 g/L sodium bicarbonate (21,602; USB, Ohio, USA), 10 % fetal bovine serum (FBS) (10,270,106; Gibco) and 1% antibiotic-antimycotic (15,240,062; Gibco). HepG2 cells were maintained at 37 °C and 5% CO_2_ in a 95 % humidified air atmosphere using AutoFlow NU-4750 Water Jacket CO_2_ Incubator (Nuaire, Minnesota, USA). ZFL were maintained at 28 °C without any supply of CO_2_ and humidity control in a Sanyo MCO175 incubator (Sanyo, Osaka, Japan) [9,26,32].

### 2.2 Chemical treatments

Stock solutions of 1 M CdCl_2_ (CAS 10108−64-2, 20,899; Sigma, St. Louis, Missouri, USA) were prepared in Nanopure water and stored at 4 °C. All chemicals were diluted with serum-free medium to the final concentrations immediately before use [7,21,27].

### 2.3 Cytotoxicity assay

The alamarBlue assay (CA92008; Invitrogen, Carlsbad, USA) was used to determine cell viability as previously reported [32]. The cells were exposed to different concentrations of CdCl_2_ for 24 h. After CdCl_2_ exposure, the alamarBlue medium was added to the cells, and the cells were further incubated for 2 h at 37 °C (for HepG2 cells) or 28 °C (for ZFL cells). Fluorescence and luminescence were then determined using a BMG CLARIOstar Microplate Reader (BMG LABTECH, Ortenberg, Germany).

### 2.4 Quantitative real-time PCR (qPCR)

Briefly, cDNA was generated from total RNA, using a method similar to the methods reported previously [32]. All qPCR amplifications and detections were performed using Premix ExTaq (RR039A; Takara, Shimogyō-ku, Japan). The DNA primers designed for use in qPCR were the same as those in our previous study [26,32].

### 2.5 Cellular metal content measurement

The procedures followed were as described in our previous studies [26,32]. In brief, the exposed ZFL and HepG2 cells were trypsinized after washing with phosphate-buffered saline (PBS) three times, resuspended and lysed in 0.03 M HNO_3_ by three “freeze-thaw’’ cycles oscillating between −80 °C and 25 °C. Metal concentrations were measured using an atomic absorption spectrophotometer (Hitachi Z2700 with Graphite Furnace). Metal content was normalized to cell number as determined by the alamarBlue assay and referenced against viable cell counts.

### 2.6 Western blot analysis

HepG2 and ZFL cells were homogenized in ice-cold RIPA buffer (89,900; Thermo Scientific, Massachusetts, USA) supplemented with Complete Protease Inhibitor Cocktail (05,892,970,001; Sigma) by triturating several times and shaking the extract 30 times. Immunoblotting was subsequently performed to detect the expression levels of CTR1 and β-actin (loading control). Proteins were loaded onto sodium dodecyl sulfate-polyacrylamide gel electrophoresis (SDS-PAGE) mini gels and transferred onto nitrocellulose membranes for incubation with the following antibodies: anti-hCTR1 (PA1−16586; Invitrogen), anti-c-Myc (9B11; Abcam, Cambridge, UK) and anti-β-actin (20,536-I-AP; Proteintech, Illinois, USA). The membranes were incubated at 4 °C overnight and then washed three times for 10 min by using Tris-Buffered Saline with 0.1 % Tween 20 Detergent (TBST) each time. After the final wash, the membranes were incubated with horseradish peroxidase-conjugated secondary antibody (1:5000). ECL (R-03,031-D25; Advansta, California, USA) substrates were used to visualize signals by chemiluminescence, and the expression levels were normalized against endogenous β-actin levels.

### 2.7 Construction of stable cell lines

Three stable cell line were generated and used in this study; an hCtr1-overexpression HepG2 cell line, a zCTR1-overexpression ZFL cell line and an hCTR1 knockdown HepG2 cell line. Each modified cell line has its own control cell line. hCTR1-overexpression HepG2 and zCTR1-overexpression ZFL cell lines were already established and are detailed in our previous study [26,32]. Briefly, hCTR1- or zCTR1-overexpression cell lines were created by transfection with Lipofectamine 3000 (L3000015; Invitrogen) to introduce linearized DNA pcDNA3.1-hCtr1 or pcDNA3.1-zCtr1 to HepG2 or ZFL cells, respectively, or the vector pcDNA3.1, as a control.

We generated hCtr1 knockdown HepG2 cells for this study using the BLOCK-iT™ Lentiviral Pol II miR RNAi Expression System with EmGFP (K493800; Invitrogen). As per the manufacturer’s instructions, we designed oligos (5′-TGCTGTGTGCAGCACTGTTTGCAGGAGTTTTGGCCACTGACTGACTCCTGCAAAGTGCTGCACA -3′ and 5′−CCTGTGTGCAGCACTTTGCAGGAGTCAGTCAGTGGCCAAAACTCCTGCAAACAGTGCTGCACAC -3′) and cloned them into the vector provided (pcDNA6.2- GW/+EmGFP-miR) to produce the plasmid pcDNA6.2-mihCtr1, which was used for knockdown of hCTR1 in HepG2 cells. The negative control plasmid pcDNA6.2-GW/miR-neg (pcDNA6.2-Neg) was supplied with the kit, and contains a sequence that does not target any gene. Eco91I (FD0394; Thermo Scientific) was used to linearize pcDNA6.2-mihCtr1 and pcDNA6.2-Neg, which were subsequently transfected into HepG2 cells. Successfully transfected HepG2 cells were selected by treatment with 2 μg/mL Blasticidin S HCl (A1113902; Gibco).

### 2.8 Immunofluorescence

HepG2 cells (10^6^ cells /well) were grown in 6-well plates on glass coverslips overnight. The medium was removed, and cells were washed three times with ice-cold PBS followed by fixation in 4% paraformaldehyde at room temperature for 10 min. After fixation, HepG2 cells were washed with ice-cold PBS twice and then permeabilized with 0.05 % Triton X-100 for 8 min. Cells were blocked with 0.5 % bovine serum in PBS. HepG2 cells were subsequently incubated with anti-CTR1 antibody and Alexa-488 anti-rabbit antibodies (1:5000) sequentially for 1 h in room temperature. The fixed cell images were recorded using a Leica TCS SP8 Confocal Microscope System (405 nm and 488 nm lasers), at 63 × magnification and pin hole = 1.0.

### 2.9 Protein structure and docking

The predicted 3D protein structures of hCTR1 and zCTR1 were generated using SWISS-MODEL [33,34]. We uploaded the. pdb file to the Metal Ion-Binding Site Prediction and Docking Server (MIB) [35], for predicting the position of the Cd^2+^-binding site. The structures (.pdb files) created by SWISS-MODEL were viewed using UCSF Chimera [36].

### 2.10 Statistical analysis

All statistical analyses and graph generation were performed using GraphPad Prism 8.0. Two-way ANOVA with multiple comparisons (Uncorrected Fisher's LSD test) was used to determine the significance (* *p* <  0.05) for the AAS experiments; one-way ANOVA was used to compare the treated groups and the controls (untreated groups). T-test was used to determine the significance (* *p* <  0.05) for the mortality value comparison. Data are expressed as the mean ± standard error of the mean of biological replicates (n = 3) unless specified otherwise.

## 3 Results

### 3.1 Cell viability and Cd^2+^ uptake in HepG2 and ZFL cells

**Fig. 1:**
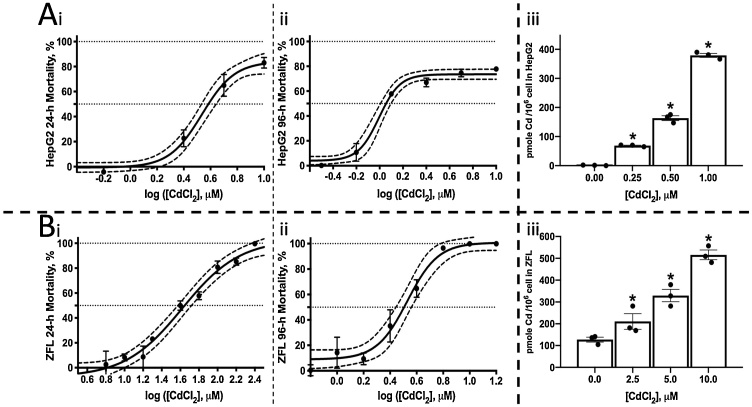
The cell viability (%) of Cd^2+^ and the intracellular Cd^2+^ levels in HepG2 and ZFL cells. The dotted lines represent the 95 % confidence level of the curve. (A) HepG2, (B) ZFL. (i) cell viability after 24 h Cd^2+^ exposure, (ii) cell viability after 96 h Cd^2+^ exposure, (iii) intracellular Cd^2+^ levels per million cells after 24 h Cd^2+^ exposure. Significant differences (one way ANOVA) compared with controls (cells not exposed to Cd^2+^) are highlighted with *.

We first examined the cell viability of HepG2 and ZFL cells exposed to various concentrations of CdCl_2_ (between 0.16 μM to 400 μM) to determine the LC_50_ values. After 24 h, the LC_50_ of HepG2 was 3.89 μM (95 % CI: 3.40–4.39 μM) (Fig. 1Ai), whereas the LC_50_ after 96 h exposure was 1.13 μM (95 % CI: 1.02–1.25 μM) (Fig. 1Aii). In the case of ZFL cells, after 24 h, the LC_50_ was 44.4 μM (95 % CI: 37.4–52.5 μM) (Fig. 1Bi), whereas after 96 h, the LC_50_ was 3.20 μM (95 % CI: 2.78–3.66 μM) (Fig. 1Bii).

We next studied intracellular Cd^2+^ levels in cells exposed to Cd^2+^ using both dosage and time course studies. The Cd^2+^ levels within exposed cells reflects Cd^2+^ absorption or uptake by the cultured cells. We selected Cd^2+^ concentrations based on the previously determined LC_50_, a concentration that results in limited cell death. As such, we determined intracellular Cd^2+^ levels after exposure to 0.25, 0.5 and 1 μM Cd^2+^ in HepG2, and after exposure to 2.5, 5 and 10 μM in ZFL cells. Intracellular Cd^2+^ levels increased in both HepG2 and ZFL cells with increasing exposure concentrations. After exposure to 1 μM Cd^2+^ for 24 h, 379 pmole Cd^2+^ was found in one million HepG2 cells (Fig. 1Aiii). In contrast, 516 pmole Cd^2+^ was found in one million ZFL cells following exposure to 10 μM Cd^2+^ for 24 h (Fig. 1Biii). Thus, for both cell lines, Cd^2+^ uptake occurred, indicating it can penetrate cell membrane by some unknown mechanism.

### 3.2 Cd^2+^ did not alter hCTR1 expression but altered its intracellular localization

**Fig. 2:**
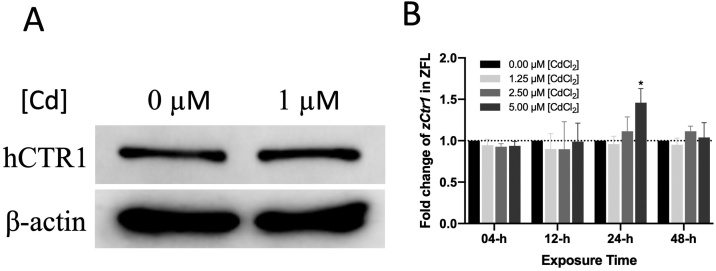
CTR1 expression levels following Cd^2+^ exposure. (A) Total CTR1 protein in HepG2 cells in the presence or absence of Cd^2+^ after 24 h. β-actin was used as a loading control. (B) The *zCtr1* mRNA levels in ZFL at various Cd^2+^ concentrations and exposure time points. *zybx1* was used as a housekeeping gene. The bars represent the geometric mean of fold differences derived from biological replicates (n = 6), and the error bars represent the geometric standard deviation. Significant differences compared with controls (cells not exposed to Cd^2+^) using ΔΔCt are highlighted with *.

We did not observe any change in hCTR1 protein levels in HepG2 cells after exposure to 1 μM Cd^2+^ for 24 h (Fig. 2A). As an anti-zCTR1 antibody was not commercially available, we did not perform western blotting to determine zCTR1 protein levels in ZFL cells. We instead determined *zCtr1* mRNA levels using real-time quantitative PCR (qPCR) to investigate whether Cd^2+^ exposure altered the expression of *zCtr1*. However, 5 μM Cd^2+^ only induced *zCtr1* expression slightly (0.46-fold increased) over 24 h (Fig. 2B). *zCtr1* expression levels remained unchanged at other low concentrations, or over both longer and shorter exposure times. Thus, Cd^2+^ does not alter CTR1 expression at either the protein or mRNA levels.

**Fig. 3:**
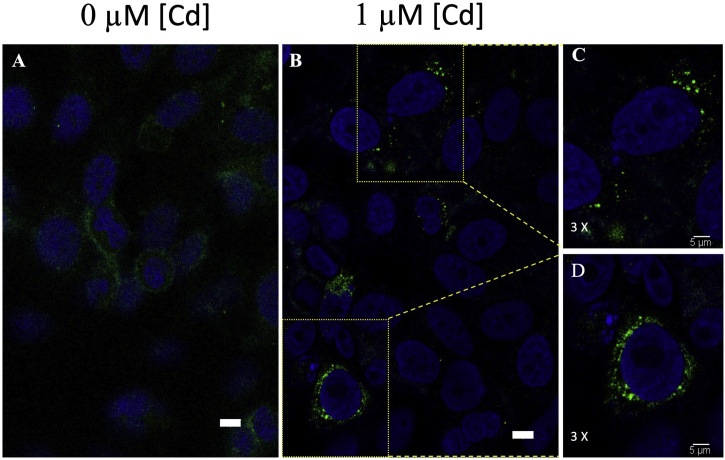
Immunofluorescence determination of hCTR1 subcellular localization in HepG2 cells in the presence or absence of Cd^2+^. Nuclei were stained with DAPI (S36973; Invitrogen), and CTR1 were probed with Alexa-488 anti-rabbit antibody. (A) Control, in the absence of Cd^2+^ exposure. Scale bar, 10 μm. (B) Following exposure to 1 μM Cd^2+^ for 24 h. Scale bar, 10 μm. (C) Zoom of HepG2 cells following Cd^2+^ exposure. 3 × zoom; scale bar, 5 μm. (D) Zoom of another image frame following Cd^2+^ exposure. 3 × zoom; scale bar, 5 μm.

We used immunofluorescence to asses any changes in CTR1 intracellular localization in HepG2 cells following Cd^2+^ exposure. It was found that CTR1 was internalized after Cd^2+^ exposure (Fig. 3), similar to what is observed following Cu exposure [32,37]. We believe that Ctr1 responded to Cd^2+^, similar to its original ligand which is Cu^2+^. If there is no Cd^2+^ or very little Cd^2+^, CTR1 could spread widely over the cell to absorb Cu. However, CTR1 is arrested in some organelles, most likely Golgi, reduced to locate in membrane and try to cut off the Cd^2+^ absorption via CTR1when the outer Cd^2+^ concentration is high. *In silico* investigations revealed that hCTR1 and zCTR1 have highly similar protein sequences (73 % identical), so we expected their structures to be similar too (Fig. S1).

According to MIB [35], Cd^2+^ could potentially bind to the 64th (Asn) and 68th (Glu) residues of hCTR1 and to the 62th (Asn) and 66th (Glu) residues of zCTR1 (Fig. S2), all of which are located in the extracellular domain. CTR1 is localized to the cell membrane in a homo-trimer form, meaning a CTR1 channel could potentially bind three Cd^2+^ ions.

### 3.3 Overexpression and knockdown of hCTR1 in HepG2 cells

**Fig. 4:**
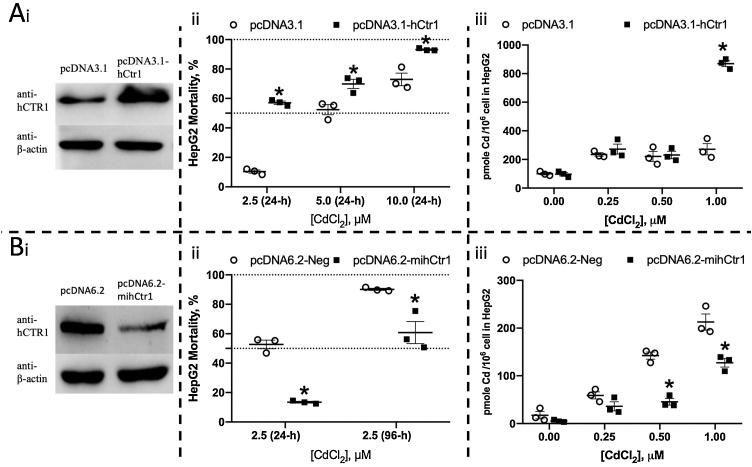
Changes of the cell viability and the content of intracellular cadmium in the HepG2 cells with overexpression or knockdown of the gene CTR1 after exposure to Cd^2+^ for 24 or 96 h. (A) HepG2 cells overexpressing hCTR1 (pcDNA3.1-hCtr1) and the control (pcDNA3.1). (B) HepG2 cells depleted of hCTR1 (pcDNA6.2-mihCtr1) and the control (pcDNA6.2-Neg). (i) hCTR1 protein levels. β-actin was used as a loading control. (ii) The cell viability following 24 h and 96 h exposure to various Cd^2+^ concentrations. (iii) Cellular Cd^2+^ content after 24 h exposure to various Cd^2+^ concentrations.

To understand the potential function of CTR1, we used a stable HepG2-overexpressing hCTR1 cell line. hCTR1 overexpression was confirmed by qPCR [26]. This stable cell line was further validated in this study for increased hCTR1 protein levels using western blotting (Fig. 4Ai). hCTR1 overexpression increased cell death compared to the control after exposure to 2.5, 5 and 10 μM Cd^2+^ for 24 h (Fig. 4Aii), as reflected in the decreased LC_50_ values at 24 h post-exposure (Fig. S3Ai). hCTR1 overexpression also increased the intracellular Cd^2+^ levels following exposure to 1 μM Cd^2+^ for 24 h compared to the wild-type cells (Fig. 4Aiii).

We also knocked down hCTR1 using miRNA to study the effect of lower hCTR1 levels on HepG2 cells. Using western blotting, we confirmed the successful creation of a cell line in which hCTR1 expression was suppressed (Fig. 4Bi). hCTR1 knockdown improved cell viability compared to the control after exposure to 2.5 μM Cd^2+^ for both 24 h and 96 h (Fig. 4Bii) and increased the LC_50_ values at both time points (Fig. S3B). hCTR1 knockdown decreased the intracellular Cd^2+^ content after exposure to both 0.5 μM and 1 μM Cd^2+^ for 24 h (Fig. 4Biii).

### 3.4 Overexpression of zCTR1 in ZFL

**Fig. 5:**
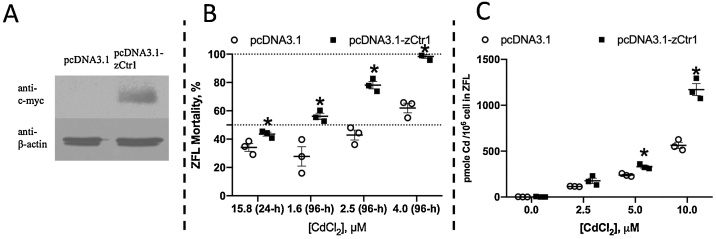
Changes of the cell viability and the content of intracellular cadmium in the ZFL cells with overexpression of the gene CTR1 after exposure to Cd^2+^ for 24 or 96 h. (A) c-Myc-linked zCTR1 protein levels. β-actin was used as a loading control. (B) The cell viability following 24 h and 96 h exposure to various Cd^2+^ concentrations. (C) Intracellular Cd^2+^ content after 24 h exposure to various Cd^2+^ concentrations.

To confirm that the functional relationship between Cd^2+^ and CTR1 was cross-species and conserved, we also studied another model organism, zebrafish. Previously, we created a stable ZFL cell line overexpressing zCTR1 [32]. As anti-zCTR1 antibody is not commercially available, we used an antibody targeting a c-Myc tag, which was included in the vector used to allow the examination of protein expression. We confirmed that the overexpression line expressed the c-Myc-tagged zCTR1 (Fig. 5A). zCTR1 overexpression was found to decrease cell viability compared to the control following exposure to various Cd^2+^ concentrations (15.8 μM in 24 h, 1.6 μM, 2.5 μM and 4.0 μM in 96 h) (Fig. 5B), and reduced the LC_50_ (Fig. S4) at both 24 h and 96 h. Moreover, zCTR1 overexpression increased the intracellular Cd^2+^ content following exposure to 5 μM or 10 μM Cd^2+^ for 24 h (Fig. 5C).

## 4 Discussion

### 4.1 Cd^2+^ toxicity to HepG2 and ZFL cells

Both HepG2 and ZFL are liver-derived cell lines but represent different species. The LC_50_ value for Cd^2+^-exposed HepG2 cells was approximately 10-fold lower than that for ZFL cells at 24 h exposure but reduced to a 3-fold difference by 96 h exposure. Based on the LC_50_ values, HepG2 cells are more sensitive to Cd^2+^ than ZFL cells, and responded in a relatively short period of time. We hypothesize that Cd^2+^ enters the cell to elicit toxic effects, rather than acting extracellularly to, for example, change the osmotic pressure. We have shown that both cell lines can accumulate Cd^2+^ intracellularly (Fig. 1).

HepG2 cells accumulated Cd^2+^ faster than ZFL cells, as the intracellular Cd^2+^ levels were similar following the exposure, but the concentration of the Cd^2+^ solution used to treat the HepG2 cells was 10-fold lower than that of the Cd^2+^ solution used to treat the ZFL cells. Thus, the differences seen in toxicity between the two cell lines was larger after only 24 h exposure due to the slower uptake of Cd^2+^ by ZFL cells. The effects of the differential uptake rates became negligible when the exposure time was extended further, with the LC_50_ values reducing from a 10-fold difference at 24 h to only a 3-fold difference by 96 h.

### 4.2 CTR1 facilitate Cd^2+^ uptake into the cells

In this study, we demonstrated that of CTR1 overexpression increased intracellular Cd^2+^ content in both cell lines and that hCTR1 knockdown reduced intracellular Cd^2+^ content in HepG2 cells, indicating that the cells were able to accumulate intracellular Cd^2+^ via CTR1. Moreover, CTR1 overexpression increased Cd^2+^-induced toxicity in HepG2 cells, whereas CTR1 knockdown decreased it.

Based on subcellular localization studies, CTR1 reacted to Cd^2+^ exposure in a manner similar to that seen during Cu^2+^ exposure [32]. The cells attempted to reduce CTR1 accumulation at the plasma membrane by arresting CTR1 intracellular transport, retaining it potentially within the Golgi network. This has the effect of reducing Cd^2+^ transport into the cell and limiting toxicity. These observations support the hypothesis that CTR1 plays a role in transporting Cd^2+^ into the cell.

The effect of CTR1 overexpression in HepG2 cells was clear from 24 h onward, whereas effects were not seen until 96 h in ZFL cells. This may be because hCTR1 transports Cd^2+^ more efficiently than zCTR1. One potential explanation lies with the incubation temperatures. HepG2 cells were grown at 37 °C, whereas ZFL cells were grown at 28 °C. It is well established that the activities of transporters and enzymes increase when temperature rises [38,39].

### 4.3 Uptake of Cd^2+^ and Cu^2+^ by CTR1

Cd^2+^ has been reported to inhibit the transcription of *Ctr1* in *Saccharomyces cerevisiae* [40], however, we did not see similar effects on CTR1 expression in ZFL and HepG2 cells. Moreover, Cd^2+^ induces *zCtr1* expression if co-exposed with Cu in zebrafish gills [28].

A freshwater fish study using *Rasbora sumatrana* (Cyprinidae) and *Poecilia reticulata* (Poeciliidae) found that Cu^2+^ was more toxic than Cd^2+^ based on their LC_50_ values [41]. In another study on *Cyprinus carpio* (common carp) larvae, Cu^2+^ was inferred to be more toxic than Cd^2+^ on the basis of growth rate inhibition [42], but the extent of the inhibition was alleviated with Cu^2+^ and Cd^2+^ co-exposure.

In a recent study on ZFL cells, co-exposure of increasing concentrations of Cu^2+^ with a constant concentration of Cd^2+^ could increase toxicity, whereas lower concentrations of Cu^2+^ did not alter intracellular Cd^2+^ levels. These results indicate that Cu^2+^ might reduce Cd^2+^ uptake [43]. Combining these observations with our findings in the present study, Cd^2+^ antagonizes Cu^2+^ accumulation via CTR1 in ZFL cells, but the effect is very limited. It has also been reported that Cd^2+^ can enter the cell via calcium channels, DMT1 and ZIP8, in zebrafish [28]. Cd did not affect the Cu accumulation in zebrafish’s gills [28]. However, Cd could inhibit on Cu absorption in *Artemia Urmiana Nauplii* [44].

It would appear that no single metal ion channel or transporter is responsible for Cd^2+^ uptake. The calcium channels DMT1, ZIP8 and ZIP14 have been studied in rat hepatocytes and enterocytes, they were also found to be related to Cd^2+^ uptake via a mechanism wherein the adsorbed Cd^2+^ becomes bound to metallothioneins (MTs) [45]. Whether Cd^2+^ uptake via CTR1 requires binding to MTs remain to be determined.

## 5 Conclusions

This study showed that CTR1 overexpression increased Cd^2+^ uptake and Cd^2+^-induced toxicity in HepG2 and ZFL cells, whereas CTR1 knockdown decreased Cd^2+^ uptake and reduced toxicity in HepG2 cells. Cd^2+^ reduced the amount of CTR1 located at the plasma membrane, clustered inside the cells, to reduce Cd^2+^ uptake and mitigate toxicity. Thus, CTR1 is responsible for the transport of Cd^2+^ into cells.

## Authors’ contributions

Material preparation, data collection and analyses were performed by Man Long Kwok, Zhen Ping Li and Tin Yu Samuel Law. The first draft of the manuscript was written by Man Long Kwok.

All authors have read and approved the final manuscript.

## Funding

This project was supported by two Direct Grants for Research from the Biological Panel in the Chinese University of Hong Kong (CUHK) to K. M. C. (4053172 and 4053240). M. L. K. is a recipient of a Postgraduate Studentship from CUHK.

## Declaration of Competing Interest

The authors declare that they have no known competing financial interests or personal relationships that could have appeared to influence the work reported in this paper.

## Figures and Tables

**Figure:**
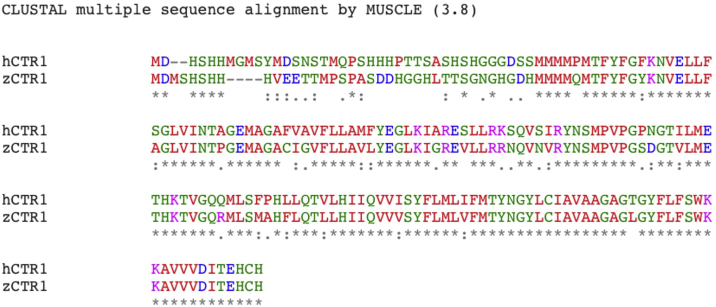


**Figure:**
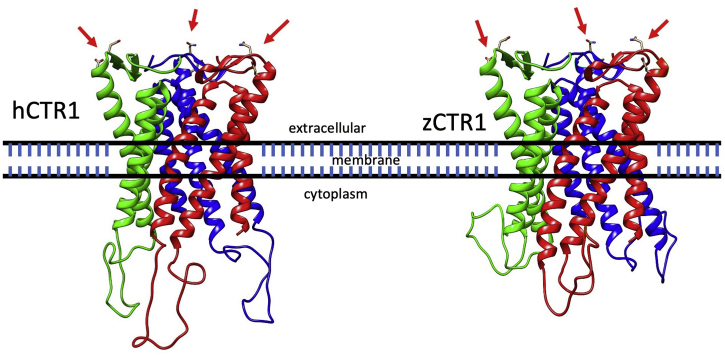


**Figure:**
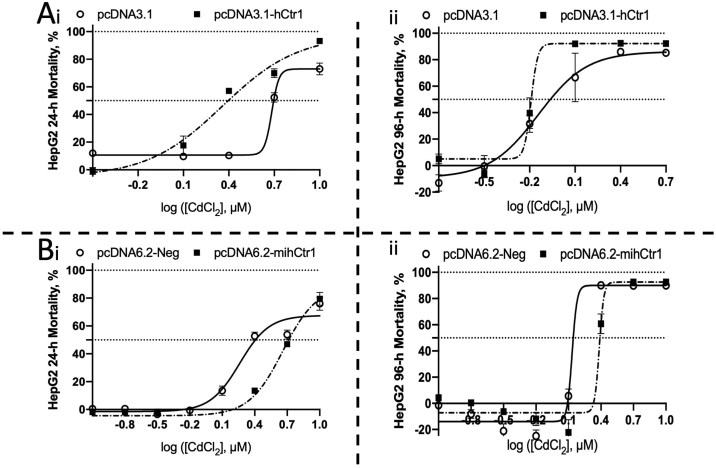


**Figure:**