# Supplementary material for: Promotion of cadmium uptake and cadmium-induced toxicity by the copper transporter CTR1 in HepG2 and ZFL cells
Source: Toxicol Rep. 2020 Nov 12;7:1564–70. doi: 10.1016/j.toxrep.2020.11.005 (PMC7695923; doi:10.1016/j.toxrep.2020.11.005)
Supplement: Supplementary file 1 [file mmc1.docx]

**Supplementary Information**

Fig. S1. Protein sequence alignments generated by MUSCLE of hCTR1 and zCTR1.

Fig. S2. 3D structure prediction by SWISS-MODEL labeled with potential metal-binding sites for both hCTR1 and zCTR1. Both structures are depicted in trimer form embedded in the plasma membrane.

Fig. S3. The cell viability curves following Cd^2+^ exposure for HepG2 cells overexpressing or depleted of hCTR1. (A) HepG2 cells overexpressing hCTR1 (pcDNA3.1-hCtr1) and the control (pcDNA3.1). (B) HepG2 cells depleted of hCTR1 (pcDNA6.2-mihCtr1) and the control (pcDNA6.2-Neg). (i) 24 h exposure. (ii) 96 h exposure.

Fig. S4. The cell viability curves following Cd^2+^ exposure of ZFL cells overexpressing zCTR1. ZFL cells overexpressing zCTR1 (pcDNA3.1-zCtr1) and the control (pcDNA3.1). (A) 24 h exposure. (B) 96 h exposure.
